# Carotenoids and Apocarotenoids in Planta: Their Role in Plant Development, Contribution to the Flavour and Aroma of Fruits and Flowers, and Their Nutraceutical Benefits

**DOI:** 10.3390/plants10112321

**Published:** 2021-10-28

**Authors:** Andrew J. Simkin

**Affiliations:** 1 School of Biosciences, University of Kent, Canterbury CT2 7NJ, UK; a.simkin@kent.ac.uk or andrew.simkin@niab.com; 2 Crop Science and Production Systems, NIAB-EMR, New Road, East Malling, Kent ME19 6BJ, UK

**Keywords:** carotenoids, apocarotenoids, biofortification, flavour, nutrition, nutraceutical

## Abstract

Carotenoids and apocarotenoids are diverse classes of compounds found in nature and are important natural pigments, nutraceuticals and flavour/aroma molecules. Improving the quality of crops is important for providing micronutrients to remote communities where dietary variation is often limited. Carotenoids have also been shown to have a significant impact on a number of human diseases, improving the survival rates of some cancers and slowing the progression of neurological illnesses. Furthermore, carotenoid-derived compounds can impact the flavour and aroma of crops and vegetables and are the origin of important developmental, as well as plant resistance compounds required for defence. In this review, we discuss the current research being undertaken to increase carotenoid content in plants and research the benefits to human health and the role of carotenoid derived volatiles on flavour and aroma of fruits and vegetables.

## 1. Introduction

**Figure 1:**
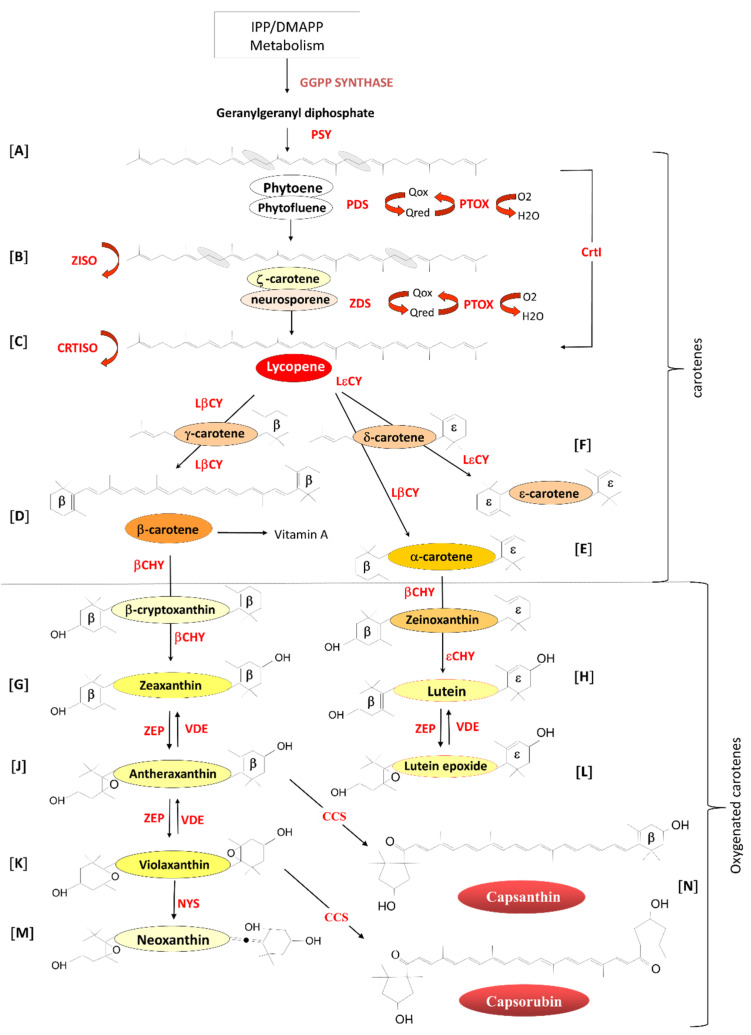
Overview of the biosynthesis of isoprenoids in plastids. PSY: Phytoene synthase. PDS: phytoene desaturase. ZDS: ζ-carotene desaturase. Z-ISO: ζ-carotene isomerase. PTOX: plastid terminal oxidase. CRTISO: carotene cis-trans isomerase. LβCY: lycopene β-cyclase. LεCY: lycopene ε-cyclase. βCHY: β-carotene hydroxylase. εCHY: ε-carotene hydroxylase. ZEP: zeaxanthin epoxidase. VDE: violaxanthin de-epoxidase. NYS: neoxanthin synthase. CCS: capsanthin/capsorubin synthase (adapted from Simkin et al. [48]. Letters A-N represent specific biosynthetic steps highlighted in the text.

Carotenoids are important natural pigments found in all plants and some bacteria, algae and fungi [1,2,3] and constitute one of the largest families of natural products, with more than 750 distinct compounds classified to date [4,5,6]. A recently published database provides information on more than a thousand carotenoid compounds, some of which still remain to be identified [7]. Structurally, carotenoids are classified into two main groups, carotenes and the oxygenated xanthophylls. Carotenes are linear, such as in the case for phytoene and lycopene, or contain a cyclized hydrocarbon ring, as seen in α-carotene and β-carotene (see Figure 1). Xanthophylls, such as zeaxanthin, violaxanthin and lutein, are oxygenated carotenes, which contain hydroxyl, epoxy or keto groups [8,9] (see Section 2).

Carotenoids have often been used as food colorants, food supplements and nutraceuticals for cosmetics industry, well as pharmaceuticals. Carotenoids have been shown to be beneficial for human health, serving as antioxidants that significantly reduce the incidence of stroke, mortality and cardiovascular disease (CVD) [10,11]. Furthermore, studies have found that a carotenoid-rich diet can reduce the risk of cervical and prostate cancers [12,13,14,15] (see Section 2.3).

Due to their often vibrant colours, carotenoids are generally considered to be simple pigments; however, carotenoids carry out important biological functions, such as the stabilisation of lipid membranes [16,17,18,19], the assembly of lipoprotein structures including plastoglobules and fibrils [20,21,22], photosynthetic light harvesting and protecting the photosystem from reactive oxygen species (ROS) mediated damage [23,24,25,26]. Deprived of the protective functions of carotenoids, the reaction centres, antenna complex and thylakoid membranes are susceptible to photo-oxidation resulting in the loss of chlorophyll and photo-bleaching. The protective function of carotenoids is so critical that any disruption in carotenoid biosynthesis is lethal to photosynthetic organisms [27,28,29,30].

In addition to their role in photosynthesis, carotenoids are cleaved both enzymatically by carotenoid cleavage enzymes (CCDs) and non-enzymatically upon exposure to light to form cleavage products that act as precursors for the formation of key regulatory molecules including strigolactones [31,32,33,34,35,36] and abscisic acid [37,38,39]. Some of these cleavage products represent key flavour and aroma molecules, such as β-ionone in fruit and flowers [40,41,42,43,44,45,46,47] (see Section 3). Others, including α-carotene, β-carotene and β-cryptoxanthin, function as precursors to the formation of vitamin A [3,48].

This review focuses on carotenoids and *Apocarotenoid biosynthesis* and their roles in plant development, the quality of food groups and their health benefits, complimenting the review published by Meléndez-Martínez et al. [6].

## 2. Carotenoids

### 2.1. Carotenoid Biosynthesis in Planta

The carotenoid biosynthetic pathway has been intensely studied since the early 1960s [9,49,50]. While the carotenoid biosynthetic genes are located in the nucleus, their precursor protein products are imported into the chloroplast where the mature proteins synthesis carotenoids [51]. In chloroplasts, carotenoids accumulate in the photosynthetic membranes in association with the photosynthetic reaction centres and light-harvesting complexes [26,52,53,54]. In fruits and flowers, petals chloroplasts differentiate into chromoplasts and carotenoids accumulate in the membranes or in oil bodies such as plastoglobules [20,22] and fibrils [21], or in other structures within the stroma.

Phytoene (Figure 1A), the first true carotenoid, is formed by the condensation of two molecules of geranylgeranyl diphosphate by the enzyme phytoene synthase (PSY; EC.2.5.1.32). Phytoene undergoes four consecutive desaturation steps catalysed by two enzymes, phytoene desaturase (PDS; EC.1.3.99.28), resulting in the formation of ζ-carotene (Figure 1B) via the intermediate phytofluene [55,56] and ζ-carotene desaturase (ZDS; EC.1.14.99.30) to form lycopene (Figure 1C), the red pigment responsible for the colour of tomatoes, via the intermediate neurosporene [57,58]. To maintain carotenoids in their trans form, ζ-carotene isomerase (Z-ISO; EC.5.2.1.12) [59] converts 9,15,9′-cis-z-carotene to 9,9′-cis-ζ--carotene via the isomerization of the 15-cis-double bond, and carotene isomerase (CRTISO; EC.5.2.1.13) [60,61,62] transforms 9,15,9′-tricis-ζ--carotene into 9,9′-dicis-ζ-carotene, 7,9,9′-tricis-neurosporene into 9-cis-neurosporene and 7,9-dicis-lycopene into all-trans-lycopene. These desaturation steps require the presence of the plastid terminal oxidase (PTOX; EC.1.10.3.11) as a co-factor [29,63,64,65,66].

Lycopene undergoes two cyclization reactions forming α- and β-carotene. Lycopene β-cyclase (LβCY; EC.5.1.1.19) introduces two β-rings to the ends of the Lycopene carbon chain forming β-carotene (β,β-carotene; Figure 1D) via the intermediate γ-carotene (β,ψ-carotene), which contains a single β-ring and one uncyclized end, known as psi (ψ) [67]. LβCY and lycopene ε-cyclase (LεCY; EC.5.1.1.18) form α-carotene (β,ε-carotene) (Figure 1E) by introducing one β-ring and one ε-ring respectively to lycopene via the intermediate δ-carotene (ε,ψ-carotene) with one ε-ring and one uncyclized ψ end [68].

In *Lactuca sativa* (lettuce), LεCY introduces two ε-rings, resulting in the formation of ε-carotene (ε,ε-carotene; Figure 1F) [69]. LεCY genes have been identified in plants, green algae and cyanobacteria (Prochlorococcus marinus), and likely arose following gene duplication of the β-cyclases and later functional divergence [70,71,72,73].

Oxygenated carotenoids are formed by the hydroxylation of the β- and ε-rings of the carotene carotenoids. β-carotene is converted to zeaxanthin (3,3′-dihydroxy-β,β-carotene) via cryptoxanthin (Figure 1G) by the action of β-carotene hydroxylase (βCHY; EC.1.14.15.24) [74,75,76,77,78], and α-carotene (β,ε-carotene) is hydroxylated by βCHY to form zeinoxanthin and then the ε-ring is hydroxylated by ε-carotene hydroxylase (εCHY; EC 1.14.99.45) to form lutein (dihydroxy-ε,ε-carotene) (Figure 1H) [79,80,81]. Lutein is essential for the assembly of the light-harvesting photosystems and plays a role in non-photochemical quenching [82,83,84,85,86,87].

Lutein has also been shown to enhance the stability of the antenna proteins [88], play a role in light harvesting by transferring energy to chlorophyll (Chl) [89] and to quench Chl triplet states in the light-harvesting complex, protecting it from photo-oxidative damage [90].

Zeaxanthin epoxidase (ZEP: EC.1.14.13.90) catalyses the epoxidation of the two hydroxylated β-rings of zeaxanthin in two steps to generate antheraxanthin (Figure 1J) and violaxanthin **(**Figure 1K; [91,92]. In high light, violaxanthin is converted back to zeaxanthin by the activity of violaxanthin de-epoxidase (VDE: EC.1.10.99.3). This inter-conversion of violaxanthin to zeaxanthin is called the xanthophyll cycle and is implicated in the adaptation of plastids to changing light conditions [93,94,95]. In a similar mechanism, ZEP and VDE catalyse the inter-conversion of Lutein to Lutein epoxide (Figure 1L) in a process first reported in green tomato fruit in 1975 [96].

The final carotenoid, neoxanthin (Figure 1M), is synthesized from violaxanthin by the enzyme neoxanthin synthase first cloned from tomato and potato (NYS: EC.5.3.99.9) [97,98]. In Capsicum annum, antheraxanthin and violaxanthin are modified by a unique enzyme, capsanthin/capsorubin synthase (CCS: EC.5.3.99.8), induced at the onset of ripening [99], resulting in the synthesis of capsanthin and capsorubin from antheraxanthin and violaxanthin, respectively (Figure 1N) [100,101]. CCS possesses 86.1% amino acid sequence similarity with the tomato βCHY, suggesting that the two genes evolved from a common ancestral form and that the CCS functional activity diverged at a later date [102,103].

### 2.2. Manipulating Carotenoid Content in Planta

Metabolic engineering has been used to generate a large number of crops with substantial increases in carotenoid content. Since carotenoid levels are determined by the rate of biosynthesis, the means of carotenoid sequestration and finally the rate of degradation, multiple avenues exist to increase carotenoid content in planta. The ‘push’ strategy uses methods to increase metabolic flux by over-expression of carotenoid biosynthesis enzymes. The ‘pull’ strategy increases carotenoid sink capacity and finally, the ‘block’ strategy seeks to reduce the rate of carotenoid turnover.

#### 2.2.1. ‘Push’ Strategies for Increasing Carotenoid Content in Planta

Using genetic engineering to increase carotenoid content in fruit and staple crops has the potential to increase the availability of carotenoid substrates for the generation of a host of important volatile and non-volatile organic compounds and important nutritional components of foods. Genetic engineering of the carotenoid biosynthesis has been shown to create high carotenoid varieties of key staple crops such as flaxseed (*Linum usitatissimum*) [104,105], wheat (*Triticum aestivum*) [106], Sorghum [107,108], canola (*Brassica napus*) [109] and rice (*Oryza sativa*) [110,111,112], and root crops such as potato (*Solanum tuberosum*) [113,114,115] and cassava (*Manihot esculenta*) [114]. In addition, work to produce high carotenoid varieties of tomato (*Solanum lycopersicum*) has been well studied [22,116,117], (Table 1).

Key staple crops such as rice (*Oryza sativa*), wheat, cassava and potato, which constitute a significant part of the diets of poorer communities, contain little or no carotenoids or carotenoid-derived compounds (CDCs). Early efforts to generate β-carotene enriched-rice (*Oryza sativa*), termed “golden rice” [110,111,112], by over-expressing multiple enzymatic steps in the pathway (Figure 1) successfully resulted in rice variety accumulating up to 18.4 µg/g of carotenoids (up to 86% β-carotene) [111]. In this instance, these authors over-expressed PSY with the expression of the *Pantoea ananatis* CrtI (EC 1.3.99.31). CrtI carries out the activities of four plant enzymes, namely PDS, Z-ISO, ZDS and CRTISO (Figure 1).

Paine et al. [111] also demonstrated that PSY was critical to maximizing carotenoid accumulation in rice endosperm (Table 1). Golden rice was engineered with the hope of combatting early death and premature blindness and caused by vitamin A deficiencies in populations that consume quantities of white rice which is known to be nutrient poor (see Section 2.3).

**Table 1:** Summary of the cumulative impacts of multiple transgenes manipulating carotenoid accumulation in crops (See Figure 1). 1-Deoxy-d-xylulose-5-phosphate synthase (*Dxs*); phytoene synthase (Psy) phytoene desaturase (Pds); lycopene β-cyclase (Lyc); *Hordeum vulgare* homogentisic acid geranylgeranyl transferase (HGGT); *Erwinia uredovora* phytoene synthase (CrtB); *Erwinia uredovora* phytoene desaturase (CrtL); *Pantoea ananatis* phytoene desaturase (CrtI); *E. uredovora* lycopene β-cyclase (CrtY); *Escherichia coli* phosphomannose isomerase (PMI); *E.coli* 1-Deoxy-d-xylulose-5-phosphate synthase (DXS).

Plant	Transgene(s)			Metabolite Analysis	Ref
*Tomato fruit*	crtB	-	-	phytoene content increased (1.6–3.1-fold). Lycopene (1.8–2.1-fold) and β-carotene (1.6–2.7-fold) were increased	[117]
	crtL	-	-	β-carotene content increased about threefold, up to 45% of the total carotenoid content	[116]
	SlPSY	-	-	phytoene content increased 135%; β-carotene increased 39%; total carotenoids increased by 25%	[118]
	AtPDS	-	-	Lycopene and β-carotene increased 31.1% and 42.8%, respectively, and phytoene decreased by up to 70%	[119]
	AtZDS	-	-	18–26% increase in lycopene in fruit	[120]
	SlLyc	-	-	Increase in total carotenoids (2.3-fold). β-carotene increased (11.8-fold), and Lycopene decreased (10-fold)	[121]
*Cassava tubers*	crtB	-	-	~15-fold increases in carotenoids (as all-trans-β-carotene) (40–60 µg/g DW compared to CN 0.5–1 µg/g DW)	[114]
	crtB	AtDXS	-	Up to 30-fold carotenoid increase (as all-trans-β-carotene) (25 µg/g DW) compared to CN 0.5–1 µg/g DW)	
*Potato tubers*	DXS	-	-	2-fold increase in total carotenoids; 7-fold increase phytoene	[122]
	crtB	-	-	Carotenoid levels reached 35 μg/g. β-carotene levels in the transgenic tubers reached ~11 μg/g DW	[115]
	crtB	AtDxs	-	37–109 µg/g DW total carotenoids (CN 8 µg/g)	[114]
	crtB	crtL	crtY	20-fold increase (to 114 µg/g DW) with β-carotene 3600-fold higher (47 µg/g DW)	[113]
*Canola seed*	crtB	-	-	50-fold increase in carotenoids with α- and β-carotene. Lutein, the predominant carotenoid in CN seeds, remained at similar levels in transgenic seeds	[109]
*Soybean*	crtB	-	-	Accumulate 845 µg/g DW of β carotene. An increase of 1500-fold compared to CN	[123]
*Wheat*	ZmPsy	ctrI	-	Increase in β-carotene from 0.81µg/g DW to 2.3–4.9 µg/g DW in the best lines	[106]
*Cavendish Banana*	MtPsy	-	-	Increase in β-carotene content from 3.1 µg/g DW in fully ripe fruit to up to 8.3 µg/g DW.	[124]
	ZmPsy	-	-	Increase in β-carotene content from 3.1 µg/g DW in fully ripe fruit to up to 9.0 µg/g DW.	
	ZmPsy	ctrI	-	Increase in β-carotene content from 3.1 µg/g DW in fully ripe fruit to up to 13.2 µg/g DW.	
*Maize*	ZmPsy	ctrI	-	Increase in β-carotene from 0.35 µg/g DW to 15–59 µg/g DW in the best lines. Up to 100-fold increase in total carotenoids	[125]
	crtB	ctrI	-	Increase β-carotene from 0.39 µg/g DW to 9.8 µg/g DW	[126]
*Rice*	NpPsy	crtI	-	β-carotene, + small amounts of lutein and zeaxanthin	[110,112]
	NpPsy	crtI	NpLyc	1.6 µg/g DW carotenoid in the endosperm	[111]
	NpPsy	crtI	-	0.8–1.2 µg/g DW (up to 68% β-carotene)	
	SlPsy	crtI	-	0.9–1.2 µg/g DW (up to 68% β-carotene)	
	CaPsy	crtI	-	1.1–4.7 µg/g DW (up to 80% β-carotene)	
	OsPsy	crtI	-	Up to 18.4 µg/g DW (up to 86% β-carotene)	
	ZmPsy	crtI	-	Up to 14.4 µg/g DW (up to 89% β-carotene)	
	ZmPsy	crtI	-	Up to 5.5 µg/g DW (up to 39% β-carotene)	[127]
	ZmPsy	crtI	AtOr	Up to 25.8 µg/g DW (up to 50% β-carotene)	
*Sorghum*	AtDxs	ZmPsy	ctrI, PMI	β-carotene levels ranged from 2.5 to 9.1 μg/g DW in the mature seeds compared to CN 0.5 μg/g DW (+10-fold)	[107]
	HGGT AtDxs	ZmPsy,	ctrI, PMI	all-trans β-carotene levels ranged from 7.3 to 12.3 μg/g DW in the mature seeds compared to CN 0.5 μg/g DW (~19-fold increase)	

*Oryza sativ (Os); Solanum lycopersicum (Sl); Capsicum annum (Ca); Arabidopsis thaliana (At); Zea mays (Zm); Narcissus pseudonarcissus (Np); Hordeum vulgare (Hv); Musa troglodytarum x acuminate (Mt).* CN = control.

#### 2.2.2. ‘Pull’ Strategies for Manipulating Carotenoid Storage in Planta

Another route to increasing carotenoid content in fruit, manipulating carotenoid storage sinks, has also been explored (Table 2). For example, the over-expression of the Or protein has been shown to result in a significant increase in carotenoid content in tomato fruit and tubers [20,22,128,129,130,131,132]. In transgenic tomato, expression of the Arabidopsis Or was shown to promote chloroplast to chromoplast differentiation inducing carotenoid accumulation at early fruit developmental [129]. Expression of AtOR under the control of an endosperm-specific promoter increased carotenoid content in corn by promoting the formation of carotenoid-sequestering plastoglobuli [133]. However, these authors showed that these increases were seen when the carotenoid pool was limited, but it had no effect when carotenoid levels where abundant [133]. In Arabidopsis, Zhou et al. [134] demonstrated that the Or protein interacts directly with PSY (see Figure 1), post-transcriptionally regulating carotenoid biosynthesis. Chayut et al. [135] demonstrated in melon (*Cucumis melo*) that CmOr is required to stabilize flux through the carotenoid biosynthetic pathway, but the increase in carotenoids is due to the inhibition of downstream metabolic turnover of β-carotene [135]. Or expression has also been shown to increase carotenoid content in the seeds of rice [127] and maise [133]. In rice, these increases in carotenoids were observed in conjunction with the over-expression of two photosynthetic genes ZmPSY and PaCrtI. When ZmPSY and PaCrtI were expressed together, rice grain accumulated up to 5.5 µg/g DW, increasing to 2.5µg/g DW when these genes were expressed along with the *AtOr* gene [127]. This is the first demonstration that a multi-gene approach, targeting both carotenoid synthesis and sequestration, has the potential to dramatically increase carotenoid levels in grain.

Furthermore, the over-expression of the pepper fibrillin in transgenic tomato showed that fibrillin proteins play a crucial role in development of plastoglobules and fibrils in differentiating chromoplast [22]. In transgenic tomato, over-expression of Fibrillin was shown to delay thylakoid loss during chloroplast to chromoplasts differentiation, increase plastoglobuli number and thereby increase the concentrations of carotenoids including β-carotene (+64%) and lycopene (+118%) [22]. These carotenoids were further shown to increase the pool of substrates for volatile formation, and fruit were shown to generate a 36% and 74% increase in β-carotene-derived volatiles β-ionone and β-cyclocitral, respectively. Furthermore, an increase in the lycopene-derived volatiles citral (+50%), 6-methyl-5-hepten-2-one (MHO; +122%) and the ζ-carotene-derived geranylacetone (+223%) were observed to be consistent with the increases in carotenoids in these fruit [22]. These results demonstrate that increasing carotenoid content in fruits, vegetables and other crops provides a substrate for the formation of important volatile and non-volatile organic compounds important to plant development, flavour and aroma.

#### 2.2.3. ‘Block’ Strategies for Manipulating Carotenoid Storage in Planta

‘Block’ strategies for increasing carotenoid content look at methods preventing carotenoid turnover by downstream enzymes. In this case, carotenoid cleavage dioxygenases (CCDs) cleave carotenoid and form a variety of apocarotenoid products playing a role in carotenoid turnover (see Section 3). Arabidopsis Carotenoid cleavage dioxygenases 1 mutants (ccd1-1) have a 37% increase in seed carotenoid content under their experimental conditions [42]. These results were confirmed by the work of Gonzalez-Jorge et al. [138], which showed the mutant ccd1-1 accumulated lutein, neoxanthin, violaxanthin and a 400% increase in β-carotene (Table 3). Carotenoid cleavage dioxygenases 4 knockout (ccd4-1) had an even higher impact on seed carotenoid levels. Total carotenoids in ccd4-1 increased by 270% and β-carotene alone increased by a remarkable 840% compared with the wild type [138]. The more significant carotenoid turnover in ccd4-1 mutants compared to ccd1-1 mutants may be linked to their subcellular location. CCD1 has been shown to be localized in the cytosol, where it may have access to carotenoids stored in the plastid envelope [40,42,139], whereas CCD4 has been shown to be localized to the chloroplast and plastoglobules [140] where carotenoids are stored, giving them easier access to these substrates. Combining ccd4-1 and ccd1-1 into a single background increased carotenoid levels in Arabidopsis seed by 360% compared with ~170% and 270% for ccd1-1 and ccd4-1 alone (Table 3).

These data suggest that CCD1 and CCD4 are important actors in carotenoid turnover and that whilst CCD4 has a more important role, likely due to its chloroplastic localisation, the two work together, and combined ccd1 and ccd4 mutants have a synergistic effect on the accumulation of carotenoids in Arabidopsis seeds. Furthermore, a mutation in ccd4 in peach (Prunus persica) was shown to result in a yellow fleshed variety due to the accumulation of carotenoids compared to the white flesh of the wild type [141].

Furthermore, work to evaluate the impact of CCDs on carotenoid turnover, authors used transgenics to knockout (KO) CCD1 or CCD4 in planta. Ohmiya et al. [142] used RNAi to silence CCD4a in Chrysanthemum (Chrysanthemum morifolium) resulted in a change of petal colour from white to yellow and Campbell et al. [143] down-regulated CCD4 in potato tubers resulting in a yellow flesh variety (Table 3).

**Table 3:** Summary of the impacts of preventing carotenoid cleavage by CCDs.

Plant	Knockout Targets		Metabolite Analysis	Ref
*Arabidopsis*	*ccd1-1*	*-*	In seeds, Carotenoids, lutein +21%, β-carotene + 86%, antheraxanthin +20%, violaxanthin +130%, neoxanthin +311% increased relative to WT	[42]
	*ccd1-1*	*-*	In seeds, Carotenoids, lutein, neoxanthin and violaxanthin increased 170% to 210%, and β-carotene 400% relative to the wild type	[138]
	*-*	*ccd4-1*	In seeds, Carotenoids, lutein +230%, violaxanthin +590%, neoxanthin +390%, and β-carotene + 840% compared with the WT	
	*ccd1-1*	*cdd4-1*	In seeds, Combining ccd4-1 and ccd1-1, antheraxanthin, and lutein levels (470, and 240% of wild-type levels, respectively), β-carotene +1710%, violaxanthin +1220%, and neoxanthin +1620 (at 1220, and 1620% of WT	
*Peach*	-	*ccd4*	Mutation in ccd4 in peach results in a yellow peach variety	[141]
*Potato*	-	ccd4 KO	Increased carotenoid content, 2- to 5-fold higher than in WT Lutein and antheraxanthin increased ~900%, violaxanthin by ~400%, and neoxanthin by ~224% in the best lines	[143]
*Chrysanthemum*	-	ccd4 KO	resulted in a change of petal color from white to yellow. During late-stage petal development, wild-type petals completely lost their carotenoids, the petals of RNAi lines contained 3 to 8 μg/g fresh weight of carotenoids	[142,144]
*Tomato*	*ccd1* KO	-	No changes observed in tomato fruit	[40]

WT = control; KO = knockout.

Furthermore, in work to evaluate the impact of CCDs on carotenoid turnover, authors used transgenics to knockout (KO) CCD1 or CCD4 in planta. Ohmiya et al. [142] used RNAi to silence CCD4a in Chrysanthemum (*Chrysanthemum morifolium*), resulting in a change of petal colour from white to yellow, and Campbell et al. [143] down-regulated CCD4 in potato tubers, resulting in a yellow flesh variety (Table 3).

Down-regulation of CCD1A and CCD1B in tomato (antisense construct) resulted in a significant reduction in the rates of emission of pseudoionone, geranylacetone and β-ionone in cut tomato fruits, volatiles generated by the 9–10(9′–10′) cleavage of lycopene, ζ-carotene and β-carotene, respectively (see Section 3.2). However, these authors did not observe significant changes in the carotenoid content of these fruits [40]. In tomato, CCD1A and CCD1B are not plastid-localized, and it is not unexpected that plants with greatly reduced CCD1 expression showed insignificant alterations in carotenoid content, given that tomato fruit accumulate a significant amount of carotenoids during ripening, and any small turnover may go unnoticed.

These areas of exploitation thus require additional research to explore the contribution of jointly manipulating ‘push’, ‘pull’ and ‘block’ mechanisms to increase carotenoid content to improve the nutritional quality of food stuffs. Carotenoids have furthermore been shown to have important health benefits when consumed as part of a balanced diet (see Section 2.3). Manipulating carotenoid biosynthesis and sequestration also offers the potential to modify the flavour and aroma of fruit, grain or leaves (see Section 3.4). However, it should be noted that blocking the carotenoids turnover could negatively impact CDCs nutritional importance. Carotenoids, via these activities of carotenoid cleavage enzymes, provide the building blocks for a number of volatile and non-volatile organic compounds of physiological importance for plant development (see Section 3).

### 2.3. ’Hidden Hunger’ and the Health Benefits of Carotenoids

A recent review by Meléndez-Martínez et al. [6] comprehensively covered the important dietary sources of carotenoids in the human diet. It has been reported that although humans had access to more than 50 carotenoids in their diet, six major carotenoids persisted in blood plasma, including the colourless carotenoids phytoene and phytofluene, and the coloured carotenoids α-carotene, β-carotene, lycopene, β-cryptoxanthin, zeaxanthin and lutein [145,146].

Carotenoids such as β-carotene, α-carotene and β-cryptoxanthin with provitamin A activity are essential in the human diet [147]. Vitamin A, also known as retinol, is an essential micronutrient and is required for growth, development and vision and is important for immune system function [148,149,150]. Vitamin A, in the form of retinal, combines with the protein opsin to form rhodopsin, a pigment containing sensory protein that absorbs light, converting it to an electrical signal, and it is required for colour vision [151]. Most people suffering from a Vitamin A deficiency are often unaware of that deficiency and show no clinical symptoms in a phenomenon often called ‘hidden hunger’ [152]. Vitamin A deficiencies are more common in areas where cereals and tubers are relied upon for the vast majority of calories consumed, as they are a poor source of provitamin A carotenoids [152].

Genetically modified maize (*Zea mays*) [125,126] (Table 1) engineered to accumulate provitamin A carotenoids has shown to be effective at increasing the stores of vitamin A in the bodies of 5- to 7-year-old children [153]. This work has shown that β-carotene-fortified maize is as effective at controlling vitamin A deficiency as taking supplements [153]. Palmer et al. [154] showed that the consumption of β-carotene from fortified maize improved the visual function of children with a vitamin A deficiency. As such, crops such as ‘golden rice’ (see Section 2.2.1) biofortified with provitamin A, engineered by European scientists with the hope of combatting premature blindness, and in extreme cases, death by vitamin A deficiencies, have great potential to improve the health of populations that that subsist on nutrient-poor white rice [48]. However, 20 year later, golden rice is not readily available to those it was intended to help. Over the past 20 years, it has been reported that tens of millions of people across Asia (Bangladesh, China and South and Southeast Asia) have gone blind or died due to these delays [155]. Some critics described golden rice as a ‘hoax’ or ‘fool’s gold’ and eventually became a key piece of what supporters have described as propaganda against GM technologies, resulting in a 20-year delay in its introduction and what supporters have described as a crime against humanity [155].

Carotenoids, such as phytoene, phytofluene, lycopene, lutein and astaxanthin, have been associated with a decreased in the risk of certain cancers, including colon [156], lung [157], and prostate cancer [158,159,160]. In elderly patients (64–75), a high intake of tomatoes, carrots and lycopene was associated with a decreased risk of prostate cancer compared to patients with a lower intake of these foods (~50% less tomatoes and 125% less carrots and a 23% lower carotenoid intake overall) [12]. For example, these authors found that patients with prostate cancer consumed 839 μg/day lycopene, 756 μg/day α-carotene and 4473 μg/day β-carotene compared to the general population with an intake of 1356, 919 and 5492 μg/day lycopene, α-carotene and β-carotene, respectively [12]. A low dietary intake of lycopene and a low plasma lycopene content have also been linked to increased mortality from oral cavity and pharynx cancer [161]. Furthermore, a study of 638 independently living 65–85 years old revealed that higher carotenoid (lycopene, lutein) serum levels and significantly higher levels of cholesterol adjusted α-tocopherol were correlated with higher cancer survival rates [162]. It has also been reported that lutein decreases the proliferation of breast cancer cells in a dose-dependent manner (6.25, 12.5, 25 and 50 μg/mL) and increases the expression of cellular antioxidant enzymes [163]. Further reports have shown that in human breast cell lines (e.g., MCF-7 or MDA-MB-235 cells) treatment with lycopene and β-carotene (0.5 to 10 μM), for 48 h and 96 h, inhibits cell proliferation [164]. Effectively, after 96h, treatment of MCF-7 cells with lycopene (2.5–10 μM) resulted in a 30% reduction in cell viability and a 20% reduction in MDA-MB-235 cell viability; however, the results obtained using MDA-MB-235 cells was only obtained with higher lycopene treatment [164]. Moreover, an additional cell line, MDA-MB-231, showed a 75% decrease in viability when treated with 10 μM lycopene after 96 h [164]. Similar results were found when these cell lines were treated with β-carotene. When treated with 10 μM β-carotene, a 40%, 30% and 70% reduction in MCF-7, MDA-MB-235 or MDA-MB-231 cell viability, respectively, was observed [164]. β-carotene at a concentration of 20 μM and has furthermore been shown to arrest the development of leukaemia cells (HL-60) by approximately 39% and significantly reduce their viability [165]. Phytofluene (10 μM) and ζ-carotene (10 μM) inhibited the cell growth of HL-60 cultures [166] (see Niranjana et al. [167] and Meléndez-Martínez et al. [145] for review).

Lycopene treatment (0–30 μM) over 0, 24, 48, and 96 h decreased the proliferation of SW480 cells 96 h after treatment with increasing effectiveness as lycopene levels increased from 10 to 30 μM [168]. Several other studies have also shown that lycopene (0–100 μM) inhibited cell growth in colorectal cancer cells (CRC) in a dose-dependent manner [169], and the proliferation of CRC was reduced by lycopene treatment to as low as 12 μM by Huang et al. [170]. A lycopene treatment of 20 mg/kg^−1^ in female Wistar rats has been shown to inhibit tumour growth [171] and protect against spontaneous ovarian cancer formation in laying hens (lycopene 26–52 mg/day/hen) [172].

It has been suggested that the preventive role of carotenoids against cancer is linked to their antioxidant activity and that regular consumption of carotenoids may alleviate oxidative stress. Lutein, zeaxanthin, and lycopene, for example, have been reported to decrease the inflammatory mediator’s production, as lycopene has been shown to have an anti-inflammatory effect on human colorectal cancer cells [168]. Lycopene and lutein have also been described as having the capacity to prevent oxidative stress-induced diseases such as cardiovascular disease in vivo (CVD) [173,174,175,176,177] and reduce LDL-cholesterol plasma levels [178]. Lutein has also been shown to reduce the risk of coronary artery disease [179] and may prevent atherosclerosis (condition where arteries become clogged with fatty deposits) development due to its anti-inflammatory and antioxidant properties and its ability to reduce the build-up of oxidized low-density lipoprotein (LDL) in the blood [180]. Lycopene has also been described as having preventive effects in atherosclerosis pathology [177]. High plasma lutein levels have also been found to reduce the risk of coronary heart disease and stroke [181] and decrease oxidative stress and apoptosis, protecting the myocardium from ischemia injury (inadequate blood supply to an organ i.e heart muscles) [176].

Carotenoids, lutein, zeaxanthin and β-carotene limit neuronal damage from free radicals, delaying the progression of neurological diseases, and dietary supplementation with lutein and zeaxanthin (2.02 mg/day) may prevent cognitive decline in those aged ≥ 60 years [182]. β-carotene has also been described as an Alzheimer’s disease antagonist [183], and high serum levels of lycopene, zeaxanthin and lutein have been linked to a reduction in mortality of Alzheimer’s sufferers [184].

It should also be noted that carotenoids have been linked to preventative roles in diabetes mellitus and osteoporosis, and numerous studies have suggested that carotenoids, including lutein and astaxanthin, could decrease age-associated decline in human skin cells and have a positive impact on the human life span (see Tan et al. [185], Rivera-Madrid et al. [186] and Milani et al. [187] for review), as well as having a beneficia effects on eye health and improving cognitive function (see Eggersdorfer et al. [188]).

The benefits noted above have suggested that increasing the levels of these beneficial carotenoids in the human diet could have a significant contribution to human health, and manipulating their metabolism would contribute greatly to this goal (see Section 2.2). Furthermore, manipulating terpenoid biosynthesis, either by increasing or decreasing specific carotenoid subsets, can lead to increases in nutritionally important compounds and flavour/aroma volatiles that could be used as a way to improve the quality in fresh produce such as tomatoes [22].

Carotenoid-derived apocarotenoids (CDCs) are formed by the oxidative cleavage of carbon–carbon double bonds in the carotenoid backbones either by carotenoid cleavage enzymes (CCDs) or via the exposure of carotenoids to ROS. Many of these apocarotenoids play key regulatory roles in plant development as growth simulators and inhibitors, signalling molecules, including as abscisic acid [37,38,189] and strigolactones [31,32,33,34,35], and have roles in plant defence against pathogens and herbivores [190]. Others act as flavour and aroma compounds in fruit pericarp, flowers and seeds [40,41,42,43,44,45,47,140,191]. The diverse variety of carotenoids (+700) means that the potential apocarotenoid products represent a significant number of natural compounds (see Section 3).

## 3. Apocarotenoids

### 3.1. Apocarotenoid Biosynthesis Is Planta

In the late 1980s, the routes for the formation of apocarotenoids were poorly understood. However, their chemical structure and studies carried out analysing volatiles produced during the ripening of mutant tomato varieties accumulating unusual carotenoids indicated that apocarotenoids were likely derived from the oxidative carotenoid cleavage [192].

**Figure 2:**
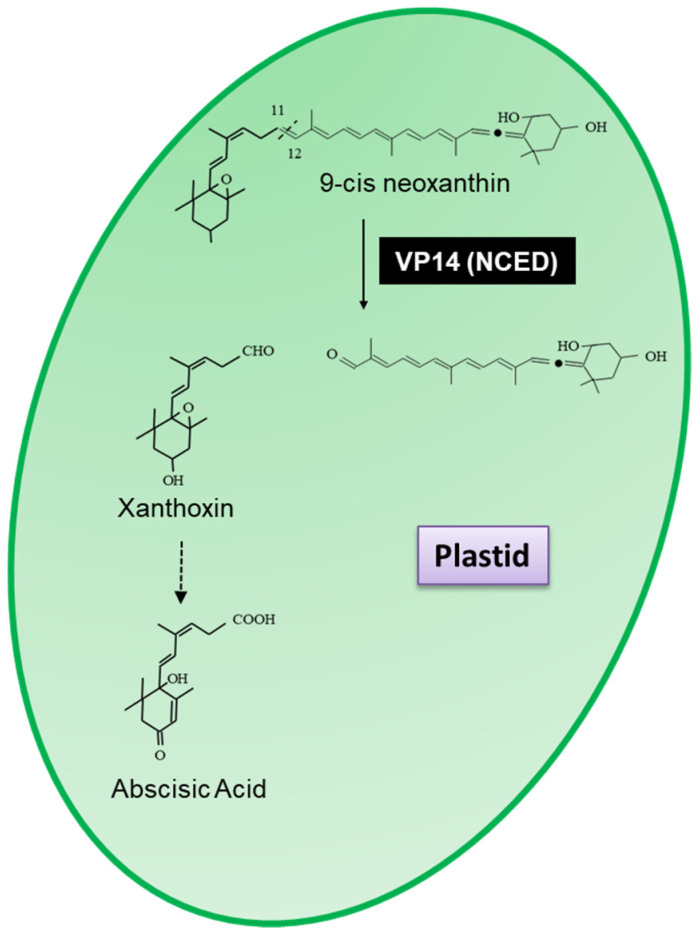
Scheme for the 11,12-cleavage reaction catalysed by VP14 (9-cis-epoxycarotenoid dioxygenase) resulting in the formation of xanthoxin, the precursor of abscisic acid.

In the years following, a family of carotenoid cleavage dioxygenases (CCDs) that are able to cleave carotenoid at an assortment of double bonds were identified [193]. The first enzyme of the CCD family was identified from *Arabidopsis thaliana* (Arabidopsis) and named VP14 (EC.1.13.11.51), which was shown to cleave 9-cis neoxanthin at the 11,12 double bond to form xanthoxin, the precursor of abscisic acid (Figure 2) [194,195].

Tan et al. [189] identified nine members of the VP14 family in Arabidopsis, five of which have been shown to cleave neoxanthin at the 11,12 double bond and have thus been renamed as neoxanthin cleavage dioxygenases (NCED2, NCED3, NCED5, NCED6(VP14) and NCED9). These enzymes have been extensively studied and are involved in the biosynthesis of the phytohormone abscisic acid (ABA). ABA regulates plant growth, development and stress responses and plays essential roles in multiple physiological processes, including leaf senescence, osmotic regulation, stomatal closure, bud dormancy, root formation, seed germination and growth inhibition among others (for review see Chen et al. and Hsu et al. [196,197]). The four remaining NCED were shown to cleave a variety of carotenoids generating a variety of (di)aldehydes and ketones [44] and were renamed carotenoid cleavage dioxygenases/oxygenases (CCD1 (EC.1.13.11.71), CCD4 (EC.1.13.11.n4), CCD7 (EC.1.13.11.68) and CCD8 (EC.1.13.11.69)).

**Figure 3:**
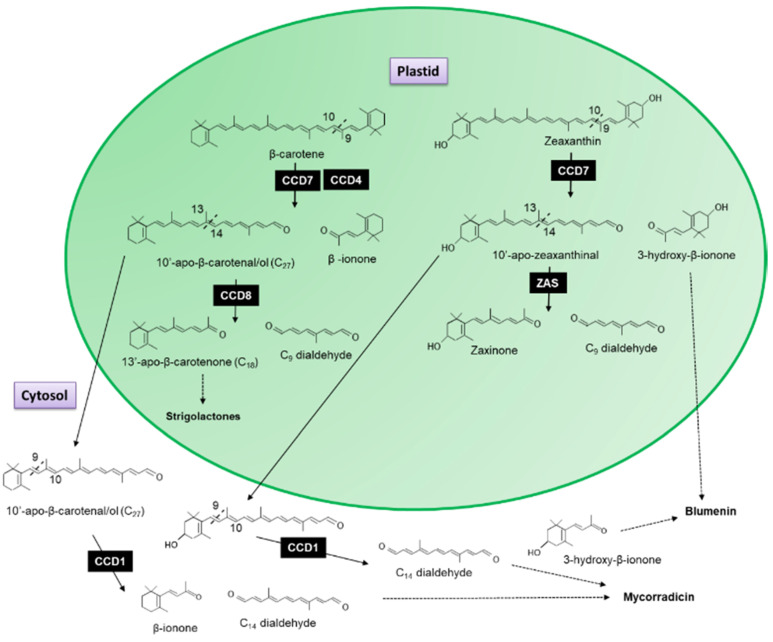
Scheme for the 9,10-cleavage of β-carotene and zeaxanthin catalysed by CCD7 and CCD4 into β-ionone and 10-apo-β-carotenal (C_27_ compound) and 3-hydroxy-β-ionone and 10-apo-β-zeaxanthinal (C_27_ compound). The 13,14 cleavage by CDD8 resulting in the formation of 9-apo-β-caroten-9-one (C_9_ dialdehyde) and the 13-apo-β-carotenone, the precursor of strigolactones. The 13,14 cleavage of 10-apo-β-zeaxanthinal by Zaxinone Synthase (ZAS) forms Zaxinone and the C_9_ dialdehyde. The C_27_ compound is cleaved by CCD1 in the cytosol into β-ionone, 3-hydroxy-β-ionone and rosafluene-dialdehyde (C_14_ dialdehyde—see Figure 4), the precursor for mycorradicin.

The recombinant CCD7 protein from Arabidopsis exhibited a 9′-10′ asymmetrical cleavage activity converting β-carotene into β-ionone (9-apo-β-caroten-9-one) and 10-apo-β-carotenal (C_27_ compound; Figure 3) [33]. When the AtCCD8 gene was expressed in *Escherichia coli* with AtCCD7, the 10-apo-β-carotenal was subsequently cleaved into 13-apo-β-carotenone and a C_9_ dialdehyde [33]. Since no cleavage activity has been associated with CCD8 when it has been expressed in carotenoid accumulation *E*. *coli* lines to date, Schwartz et al. [33] concluded that CCD8 functions as an apocarotenoid cleavage enzyme working sequential with CCD7 as the first steps in the formation of 13-apo-β-carotenone.

### 3.2. Carotenoid Cleavage Dioxygenase 1 (CCD1) Enzymes Cleave a Broad Category of Carotenoids and Apocarotenoids at Multiple Double Bonds in the Cytosol

It has been shown in the literature that this sequential cleavage is the origin of the biosynthesis of strigolactones, a new class of plant hormones essential for plant development (Figure 3; for review see [36,198,199,200]). The recently characterized Zaxinone Synthase (ZAS), previously classed as CCD8b, cleaves 3-OH-all trans-β-apo-10′-carotenal (apo-10′-zeaxanthinal), the C27 cleavage product of zeaxanthin into zaxinone (3-OH-all-trans-apo-13-carotenone), a metabolite that regulates strigolactone biosynthesis in rice and the C_9_ dialdehyde [201]. In contrast, CCD1 (Section 3.2) and CCD4 (Section 3.3) enzymes have been shown to catabolize a variety of carotenoids and produce volatile and non-volatile apocarotenoids, which are important for the aromas and flavours of flowers and fruits. The following sections of this review will focus exclusively on these two CCDs.

The majority of CCDs/NCEDs have been shown to reside within plastids. The one exception is CCD1, which acts in the cytosol to generate C_13_ and C_14_ apocarotenoids [40,42]; however, we cannot rule out a tight association with the outer envelope, as has been reported, for the tomato hydroperoxide lyase [202], which would potentially give CCD1 access to carotenoids stored in the envelope [40,42]. Significant amounts of β-carotene have been identified in the outer envelopes of spinach (*Spinacia oleracea*; [203]) and pea (*Pisum sativum*; [204]) chloroplasts, where they have been reported to stabilize chloroplast membranes [16].

Orthologs of AtCCD1 have been identified in a number of species, including tomato (*Solanum lycopersicum)* [40], strawberry (*Fragaria vesca*) [205], petunia (*Petunia hybrida*) [41], pepper (*Capsicum annum* L.) [206], coffee (*Coffea canephora/C. arabica)* [46,191], carrot (*Daucus carota* L.) [207], rice (*Oryza sativa*) [208], melon (*Cucumis melo*) [209], fig (*Ficus carica*) [210], grape (*Vitis vinifera*) [211,212,213], rapeseed (*Brassica napus*) [214], and roses (*Rosa damascena*) [215]. CCD1 has been shown to cleave both cyclic and acyclic carotenoids [40,44,208,216] forming apocarotenoid aldehydes and ketones, indicating that CCD1 may have a more complex reaction tunnel than other CCDs that cleave either cyclic carotenoids or apocarotenoids alone. Using AtCCD1, beta-apo-8′-carotenal as a substrate, isotope labelling experiments have shown that these cleavage activities arise due to a dioxygenase mechanism [217] requiring only Fe^2^ as a cofactor.

**Figure 4:**
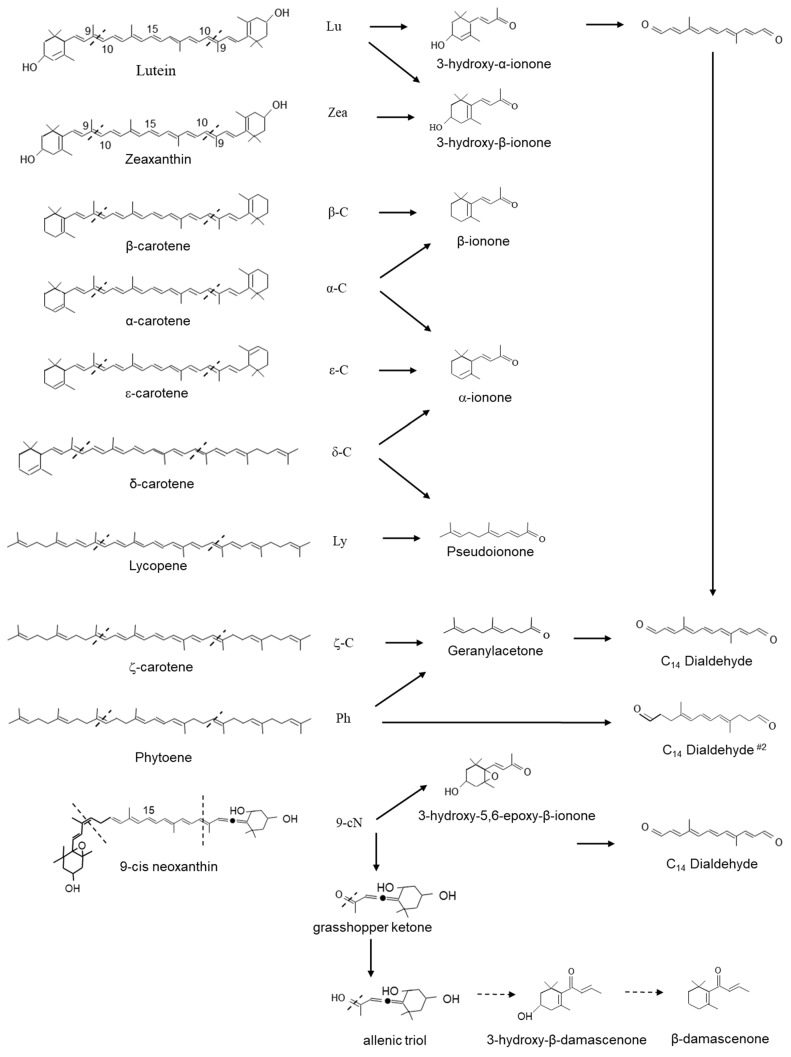
Scheme for the 9,10(9′,10′,) reactions catalysed by the recombinant CCD1 proteins with various substrates. C_14_ rosafluene-dialdehyde (4,9-dimethyldodeca-2,4,6,8,10-pentaene-1,12-dial). CCD1 activities are carried out in the cytosol.

Recombinant CCD1 enzyme and assayed multiple carotenoid substrates in vitro and characterized the cleavage products by thin-layer chromatography and HPLC. CCD1s have been shown to symmetrically cleave the 9,10(9′,10′) double bonds of a large category of carotenoids to form two C_13_ compounds and a central C_14_ rosafluene-dialdehyde (4,9-dimethyldodeca-2,4,6,8,10-pentaene-1,12-dial) (Figure 4) [40,44,218] In assays containing lutein, zeaxanthin and β-carotene, 3-hydroxy-α-ionone (3-hydroxy-9-apo-α-caroten-9-one); 3-hydroxy-β-ionone (3-hydroxy-9-apo-β-caroten-9-one) and β-ionone are formed, whereas α-carotene led to the production of both β-ionone and α-ionone and ε-carotene formed α-ionone (9-apo-α-caroten-9-one) (Figure 4).

CCD1 has also been shown to cleave nonlinear carotenoids, δ-carotene yields α-ionone (9-apo-α-caroten-9-one) and pseudoionone (6,10-dimethyl-3,5,9-undecatrien-2-one); lycopene yields pseudoionone. Several linear carotenoids, including phytoene and ζ-carotene, are thought to be the precursors of geranylacetone (6,10-dimethyl-5,9-undecatrien-2-one), an important flavour volatile in tomato fruit, as well as precursors for a second C_14_ dialdehyde (4,9-dimethyldodeca-4,6,8-triendial). Finally, in assays containing violaxanthin or neoxanthin, 5′6-epoxy-3-hydroxy-β-ionone (5,6-epoxy-3-hydroxy-9-apo-β-caroten-9-one) was formed.

In assays containing neoxanthin, the asymmetric cleavage yielded a C_27_ allenic-apocarotenal and the C_13_ grasshopper ketone (3,5-dihdroxy-6,7-didehydro-9-apo-β-caroten-9-one). The grasshopper ketone is postulated to be the precursor for the formation of the potent odorants β-damascenone (1-2,6,6-trimethyl-1,3-cyclohexadien-1-yl-2-buten-1-one) and 3-hydroxy-β-damascenone (3-hydroxy-1-2,6,6-trimethyl-1,3-cyclohexadien-1-yl-2-buten-1-one) (Figure 4) [219,220]. β-damascenone has been shown to be formed from 9′-cis-neoxanthin by peroxyacetic acid oxidation and two-phase thermal degradation without the involvement of enzymatic activity [220]. Skouroumounis et al. [221] studied the possible hydrolytic pathway of β-damascenone and suggested formation and determined that allenic triol was the key intermediate.

**Figure 5:**
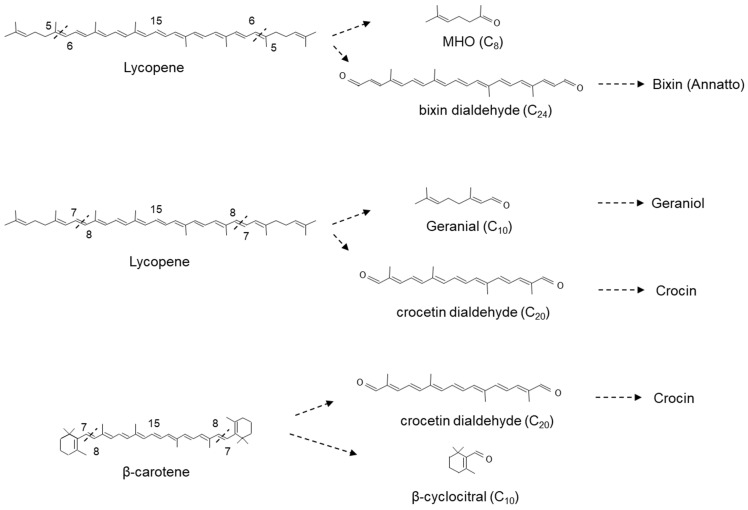
Scheme for the 5,6(5′,6′,) and 7,8(7′,8′,) reactions catalysed by the recombinant carotenoid cleavage deoxygenases (MHO; 6-methyl-5-hepten-2-one) carried out by CCD1 in the cytosol.

CCD1s from tomato and maize (*Zea maize*) have also been shown to cleave the 5,6(5′,6′) double bonds of lycopene in vitro generating the apocarotenoid 6-methyl-5-hepten-2-one (MHO; Figure 5) [216]. Furthermore, Ilg et al. [208], using both in vitro and in vivo assays, demonstrated that the activity of OsCCD1 converts lycopene into pseudoionone (Figure 4) and MHO (Figure 5), suggesting that the 7,8(7′,8′) double bonds of acyclic carotenoid ends constitute a novel cleavage site for the CCD1 plant subfamily. Carballo-Conejo et al. [222] also identified a CCD1 lycopene-specific 5,6(5′,6′)-cleavage dioxygenase (BoCCD1-1) from *Bixa orellana*, responsible for the formation of bixin dialdehyde and MHO [223,224]. Bixin dialdehyde is the precursor for the formation of the dye bixin/annatto (Figure 5; see Section 3.6.1). BoCCD1-1 gene expression correlated with bixin accumulation in *B*. *orellana* [224], suggesting that BoCCD1-1 and its homologue BoCCD1-2 could be involved in the cleavage of lycopene in seed to form bixin. However, data from a study by Cárdenas-Conejo et al. [222,223] indicated that although BoCCD1-1 is expressed in immature seed, it is also expressed in green tissue (leaf), and BoCCD1-2 was preferentially expressed in leaf. These authors also identified two additional CCD1′s, BoCCD1-3 and BoCCD1-4, which were shown to be expressed in immature seeds at 1.5-fold and 10-fold the levels found in leaf, respectively suggesting that BoCCD1-3 and BoCCD1-4 are more likely involved in the cleavage of lycopene to form bixin dialdehyde in the seed (Figure 5).

Meng et al. [225] showed that VvCCD1 also cleaved β-carotene at the 7,8(7′,8′) position into β-cyclocitral, an important flavour and aroma compound in planta. Interestingly, OsCCD1 was also shown to cleave the 7,8(7′,8′) double bonds of lycopene to form geranial (Figure 5) [208]. In the medicinal plant *Catharanthus roseus*, the formation of geraniol (isomer of geranial) from geranyl pyrophosphate by geraniol synthase [226] is a key step in the formation of a number of economically important monoterpene indole alkaloids (MIA). Several of these MIA, such as vinblastine and vincristine, are valuable therapeutic compounds (anticancer drugs: [227]). CCD1 represents a possible alternate route in the generation of geraniol in planta.

CCD1 has also been shown to cleave apocarotenoids generated by the asymmetric cleavage of a carotenoid. *Medicago truncatula* CCD1 antisense plants have been shown to accumulate 10′-apo-β-carotenal/ol (C_27_) in root material [228]. This C_27_ dialdehyde is generated by the asymmetric 9′10 cleavage of β-carotene by CCD7, which is subsequently cleaved by CCD8 to form 13-apo-β-carotenone, the precursor of strigolactones (Figure 3). This indicates that (1) CCD7 result in the accumulation of 10′-apo-β-carotenal/ol, possibly due to a low turnover by CCD8 in the strigolactone pathway; and (2) that CCD1 may act to mop up apocarotenoid generated by previous reactions. Such a role for CCD1 has been previously hypothesized (for review, see Floss et al. [229]).

The multisite cleavage of lycopene by CCD1 enzymes may be linked to the absence of a terminal ring structure found on the cyclic and oxygenated carotenoids (see Figure 1). With no ring, linear carotenoids such as lycopene may penetrate deeper into the reaction tunnel compared to cyclic carotenoids with no stop measure to prevent it. This may well result in a random cleavage pattern and the generation of multiple products from a single linear substrate (Figure 4 and Figure 5). The aldehydes and ketones generated by the activity of CCD1 enzymes represent important flavour and fragrance compounds themselves (Figure 4 and Figure 5) and act as substrates for the formation of others [40,216,230,231] (see Section 3.5).

Finally, we also cannot exclude photooxidation as an additional mechanism for the formation of 9′10(9′10′) CDCs β-ionone, pseudoionone, geranylacetone or any of the 5,6(5′6′) and 7,8(7′8′) CDCs generated by the activity of CCD1. It should be noted that the formation of β-ionone from β-carotene by free radical-mediated cleavage of the 9–10 bond has been demonstrated in vitro [232]. In pepper leaves, natural oxidative turnover accounts for as much as 1 mg of carotenoids day-1 g-1 DW [233]. During tomato fruit ripening, carotenoids concentration increases by 10- and 14-fold, mainly due to the accumulation of lycopene [234]. Given the overall quantity of carotenoids that accumulate during fruit ripening, the rates of CDC emission remain very low.

Although various CCD1s have been shown to cleave multiple double bond sites, certain species of plants or specific tissues appear to favour one activity over another, explaining why some of these final products are only detected in some plants or some organs.

### 3.3. Carotenoid Cleavage Dioxygenase 4

**Figure 6:**
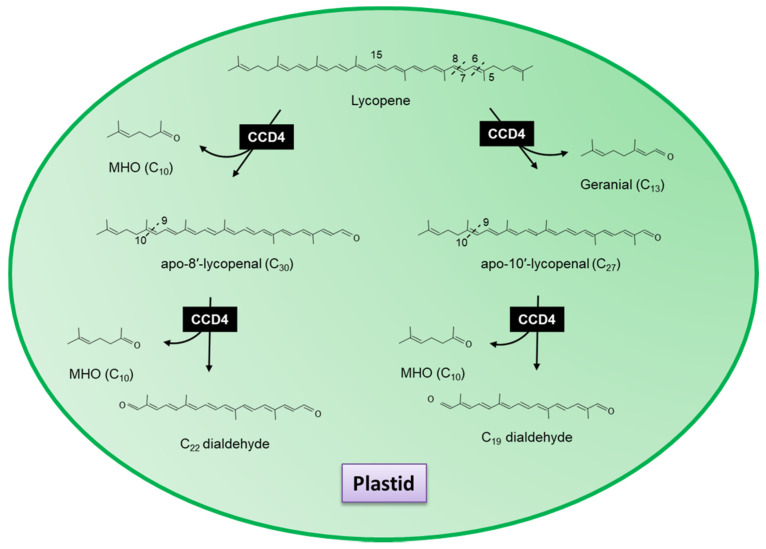
Scheme for the 5,6(5′,6′,) and 7,8(7′,8′,) cleavage of lycopene by CCD4 (MHO; 6-methyl-5-hepten-2-one) and the 9,10(9′,10′,) cleavage of the generated apocarotenoids in a second step.

Like CCD1, a common feature of CCD4 subset is a 9–10 or 9–10/9′–10′ cleavage activity [140,235]; however, unlike CCD1, CCD4 is localized to the plastid where it has been detected in the plastoglobules [140]. Plastoglobules have been identified as a site of carotenoid cleavage by a functionally active CCD4 with β-carotene, lutein and violaxanthin being the principle substrates of CCD4 in vivo [236]. Huang et al. [235] cloned CCD4 cDNAs from rose (*Rosa damascena*, RdCCD4), chrysanthemum (*Chrysanthemum morifolium*, CmCCD4a), apple (*Malus domestica*, MdCCD4) and osmanthus (*Osmanthus* *fragrans*, OfCCD4) and expressed them along with AtCCD4 in *Escherichia coli* engineered to accumulate carotenoids [237]. No cleavage products were observed for any of the five CCD4 genes when they were co-expressed in *E*. *coli* strains that accumulated either cis-ζ-carotene or lycopene. Additional trials using β-carotene as a substrate showed that CmCCD4a and MdCCD4 both cleaved the 9–10 double bond of β-carotene to yield β-ionone; however, OfCCD4, RdCCD4, and AtCCD4 were almost inactive towards this substrate. In the case of RdCCD4 and AtCCD4, the formation of β-ionone was observed in the presence of an apocarotenoid substrate, 8′-apo-β-caroten-8′-al (Figure 6), while this apocarotenal was barely degraded by MdCCD4, OfCCD4 or CmCCD4a [235]. It has also been suggested that CCD1 cleaves 10-apo-β-carotenal, a C_27_ compound generated by the activity of CCD7 (Figure 3), suggesting that CCDs also act to further catabolize down-stream products of other CCDs [229]. From the published data, it also appears that two individual classes of the CCD4 subset exist in planta. Sequence analysis showed that RdCCD4 and AtCCD4 contain no introns, whilst MdCCD, OfCCD4 and CmCCD4a contain one or two introns [142,235]. It’s interesting to note that the two intronless CCD4s displayed apocarotenoid cleavage dioxygenase activity (ACD) rather than the carotenoid cleavage dioxygenase activity (CCD) observed for the two of the three CCD4s containing introns.

In Chrysanthemum ‘Jimba’ (*Chrysanthemum morifolium*), CmCCD4a contributes to the development of white petals by cleaving carotenoids into their apocarotenoid products [142]. RNAi interference of CmCCD4a resulted in the development of pale-yellow petals due to the accumulation of carotenoids in the petal tissue [144]. Brandi et al. [238] found that CCD4 contributed to the colour of peach flesh and aroma profile, white-fleshed mutants had both a lower carotenoid content and a higher apocarotenoid aroma concentration compared to non-CCD4 expressing yellow flesh peaches, demonstrating the strong link between carotenoids and carotenoid derived aroma volatiles.

**Figure 7:**
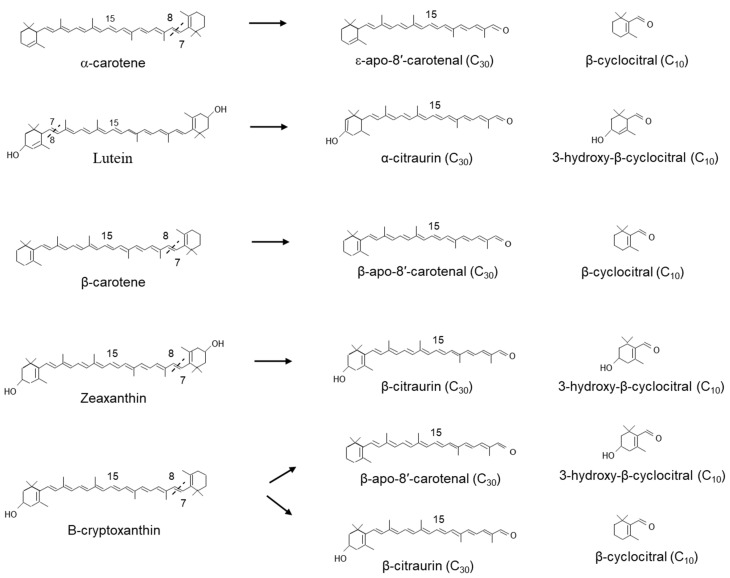
Scheme for the 7,8(7′,8′,) reactions catalysed by the recombinant carotenoid cleavage deoxygenase 4 in the plastid.

In Satsuma mandarin (*Citrus unshiu*), CitCCD4 converts zeaxanthin into 3-hydroxy-β-cyclocitral and the C_30_ apocarotenoids β-citraurin (3-hydroxy-β-apo-8′-carotenal), which is responsible for the reddish colour in the peel. CitCCD4 was also shown to use β-cryptoxanthin as an alternate substrate (Figure 7). However, CitCCD4 cleavage of β-cryptoxanthin generates two possible C_30_ apocarotenoids: β-apo-8′-carotenal or β-citraurin, although their relative abundance may indicate that the reaction favours the formation of β-apo-8′-carotenal. In the same experiments, CitCCD4 showed no activity with the substrates, lycopene, α-carotene, β-carotene or violaxanthin [239].

Related work by Rodrigo et al. [240] in the Washington Navel sweet orange (*C*. *sinensis* L. Osbeck), Clemenules mandarin (*C*. *clementina*), In silico data mining identified five CCD4-type genes in Citrus [240]. One of these genes, CCD4b1, was expressed in different Citrus species in a pattern correlating with the accumulation of β-citraurin. In contrast to the activity identified for CitCCD4, CCD4b1 was also shown to cleave β-carotene into β-apo-8′-carotenal and β-cyclocitral (Figure 7); α-carotene into one single C_30_ product, ε-apo-8′-carotenal and β-cyclocitral. When lutein was used as a substrate, only α-citraurin (3-OH-8′-apo-ε-carotenal) was identified [240], suggesting that 3-hydroxy-β-cyclocitral is also formed. In this instance, Rodrigo et al. [240] showed that CCD4b1 cleaves carotenoid structures with an ε-ring but only on the extremity containing the β-ring. These C_30_ products of lutein, α-carotene and lycopene are not detected in Citrus extracts, which is not unexpected, as lutein and α-carotene are typical only found in green fruits (see [241,242,243]).

When lycopene was used as a substrate, CCD4b1, two different apocarotenoids, apo-10′-lycopenal (C_27_) and apo-8′-lycopenal (C_30_), were identified to have derived from the 5,6 and 7,8 cleavage, respectively (Figure 6). CCD4b1 has also been shown to cleave linear apocarotenoids apo-8′-lycopenal and apo-10′-lycopenal at the 5,6 double bonds generating the C_22_ and C_19_ dialdehydes (Figure 6) [240]. These data show that the absence of the ionone ring can substantially alter the cleavage position, as has been suggested for CCD1.

MdCCD4 (*Malus domestica*), CmCCD4a (*Chrysanthemum morifolium* Ramat), RdCCD4 (*Rosa damascena*), OfCCD4 (*Osmanthus fragrans*) and AtCCD4 (*A*. *thaliana*) were all detected in their respective flowers. The expression levels of CCD4 in rose flowers were 42 times higher than those in leaves, indicating that CCD4s may play integral roles in the aroma profile of flowers [244].

### 3.4. Novel Carotenoid Cleavage Dioxygenases

**Figure 8:**
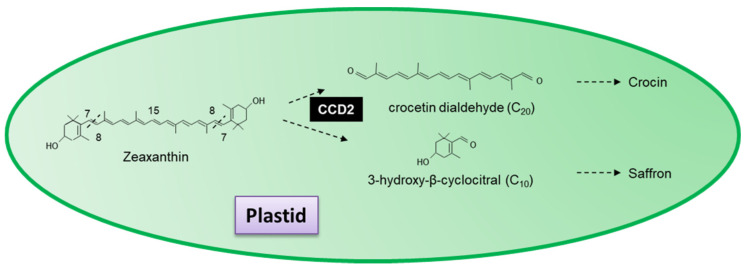
Scheme for the 7,8(7′,8′,) reactions catalysed by the recombinant carotenoid cleavage deoxygenases 2 in *Crocus sativus*.

In addition to the nine carotenoid cleavage dioxygenases initially identified (Section 3.1), authors have also identified a group of novel cleavage dioxygenases with specific activities. CCD2 is a novel carotenoid cleavage dioxygenase from *C*. *sativus* that catalyses the first dedicated step in saffron and crocin biosynthesis [139]. Localized in the plastid, CCD2 sequentially cleaves zeaxanthin at the 7,8(7′,8′) forming 3-hydroxy-β-cyclocitral and crocetin dialdehyde, the precursor for the formation of crocin and the spice saffron (Figure 8; see Section 3.6.2) [139,245]. Ahrazem et al. [245] demonstrated that CsCCD2 requires a 3-hydroxy-β-ring and does not use β-carotene or lycopene as a substrate. Crocetin dialdehyde has previously been shown to accumulate in the flowers of *Jacquinia angustifolia* [246] and the roots of *Coleus forskohlii* [247].

A second novel carotenoid cleavage enzyme, the *Zea maize* CCD10a, cleaved neoxanthin, violaxanthin, antheraxanthin, lutein, zeaxanthin and β-carotene in planta at 5,6(5′,6′) and 9,10(9′,10′) positions, generating MHO (6-methyl-5-hepten-2-one) and the C_13_ apocarotenoids, geranylacetone, α-ionone and β-ionone [248]. ZmCCD10 over-expression and down-regulation led to alterations in the expression of phosphate starvation response regulators (PHR1) and phosphate transporters, whereas down-regulation of CCD10a made plants susceptible to Pi deficiency, over-expression in Arabidopsis enhanced plant tolerance to phosphate limitation [248], further demonstrating that CCD-generated apocarotenoids have important regulatory functions in planta. Finally, Wang et al. [201] have reported that CCD10 is found in mycorrhizal plants only. As C_13_ apocarotenoids promote arbuscular mycorrhizal symbiosis (See Section 3.6.2 [228,249,250]); over-expression of CCD10 under low phosphate increases C_13_ formation, which may promote phosphate acquisition via the mycorrhiza fungal pathway (see [248]).

### 3.5. Apocarotenoids Are Important to Flavour and Aroma

Terpenoid flavour volatiles are generally present in plants at relatively low levels, but possess strong effects on the overall human appreciation of the flavour of [192,230,251,252,253,254,255,256]. These CDCs are considered to be among the most significant contributors to the flavour and aroma of many fruits and vegetables. For example, α-ionone, β-ionone, β-cyclocitral and β-damascenone have been estimated to contribute as much at 8% to the aroma profile of Valencia orange juice and as much at 78% of the total floral aroma [257]. Peak levels of β-ionone and geranylacetone emissions from ripe tomato fruit have previously been calculated to be 1.25 pg/g fw^−1^ h^−1^ and 40 pg/g fw^−1^ h^−1^, respectively [230]. Although found in low concentrations compared to other more abundant volatiles such as cis-3-hexenal and hexenal, β-ionone and geranylacetone have much lower odour thresholds, 0.007 nL/L^−1^ and 60 nL/L^−1^, respectively [230]. These odour thresholds are more than 10,000-fold lower than other flavour contributing volatiles; thus, these carotenoid-derived volatiles α-ionone, β-ionone, β-cyclocitral and β-damascenone have the potential to greatly impact aroma and flavour, even at low concentrations. Bladwin et al. [230] determined that β-ionone is the second most important volatile contributor to tomato fruit flavour. The major volatile present in lycopene-containing tomatoes (and watermelons) were geranial, MHO, pseudoionone and geranylacetone, seemingly derived from lycopene [258]. β-ionone and β-cyclocitral were identified in both tomato and watermelon fruits containing beta-carotene. Furthermore, α-ionone was detected only in an orange-fleshed tomato mutant *Delta,* that accumulates ζ-carotene [258].

β-ionone has also been identified as one of the major components of henna leaves (*Lawsonia inermis* L.) [259], melon [209] and as a constituent of a number of Moroccan herbs (*Montpellier cistus*, *Myrtus communis*, *Cistus ladanifer*) [260], and Yahyaa et al. [207] identified β-ionone in orange and purple carrot (*Daucus carota*) roots. β-ionone has also been shown to be an important contributor to fragrance in the flowers of *Boronia megastigma* [261], *Petunai hybrida* [41] and *Rosa damascene* [215] (see Paparella et al. [262] for review).

β-damascenone, which was first identified in the oil of the Bulgarian rose (*R*. *damascena*) [263], has been described as a potent odorant with an odour threshold of 2 ppt in water [231]. β-damascenone is one of the most ubiquitous natural odourants, commonly occurring in processed foodstuffs and beverages, where it has also been shown to contribute to the aroma profile of black tea [264,265], tomato [252,266], apples [267], grapefruit juice [268] and more. It has also been reported to be key component of alcoholic beverages, including wines [269,270], apple brandy [271] and beer [272,273], as well as a primary odorant in Kentucky bourbon [274]. β-damascenone has also been identified in raw coffee beans [275], which was not unexpected given the previous identification of carotenoid in raw coffee beans [191]; however, during the roasting process, β-Damascenone increased strongly [275].

Another group of volatiles synthesised by CCD1 and CCD1-like enzymes, MHO [216], and β-cyclocitral [225] are associated with tomato-like flavour [276] and sweet floral aroma [277] of tomato fruit and contributes a sweet/citrus aroma to tomato [277]. The CCD1-derived geranylacetone and pseudoionone [40] have also been identified in tomato and contribute to the overall aroma profile. Pseudoionone has been described as having a balsamic-type odour and a floral-type flavour, and geranylacetone has been described as having a floral-type odour and floral-type flavour.

Geranylacetone, α-ionone, β-ionone, β-cyclocitral and β-damascenone were all found in mango fruit [278]. Interestingly, mango fruit also contained geranial. Whether this accumulation is related to the cleavage of lycopene by CCD1 (Figure 5) or through the activity of an endogenous geraniol synthase is unknown. As noted above, geranial has also been identified in the headspace of red tomato fruit [258]. The distillation of apple brandy was also shown to enhance the concentration of two carotenoid-derived flavour compounds, β-damascenone and β-cyclocitral [271], and MHO has been shown to be present as a component of the floral scent of 50% of all flowering plants analysed [279], and β-ionone contributes to the aroma profile of petunia flowers [41]. Baldermann et al. [280] functionally characterized CCD1 from *Osmanthus fragrans* Lour and found it cleaved α- and β-ionone, contributing to the aroma of flowers.

### 3.6. Apocarotenoids Are Important Therapeutical Compounds

#### 3.6.1. Bixin

Bixin is located in the seeds of a tropical perennial achiote tree (*Bixa orellana*) grown in Central and South America, India and East Africa. It contains about 5% pigment w/v, of which 70–80% is bixin, and it is extracted to form annatto. Annatto is a commercially important natural yellow-orange-red pigment used as a dye in dairy and bakery products, vegetable oils and drinks [281,282]. Bixin dialdehyde, the precursor for the formation of bixin/annatto, is formed by the 5,6(5′,6′)-cleavage of lycopene (see Section 3.2; Figure 5) and is in increasing world demand for use as a natural food dye. Furthermore, bixin has also been described as having anti-cancer properties towards osteosarcoma, anaplastic thyroid, breast, colon, prostate and papillary thyroid cancers [283] as well as various potent pharmacological activities, including antioxidant and anti-inflammatory properties. Moreover, it has been described as a promising candidate for the treatment of Multiple sclerosis (MS), an autoimmune and degenerative disease, due to its ability to prevent neuroinflammation and demyelination in experimental autoimmune encephalomyelitis in mice, primarily by scavenging ROS [284]. Bixin has been shown to restore the sensitivity of human melanoma A2058 cells to chemotherapy and have an antiproliferative (IC_50_ = 34.11–48.17 µM) and anti-migratory effects [285]. The IC_50_ is a quantitative measurement of how much of a drug, or substance, is needed to inhibit a biological activity or process by 50%.

#### 3.6.2. Saffron and Crocetin

*Crocus sativus* L. is a perennial, stemless herb cultivated in Iran, Spain, India and Greece. Saffron, considered the world’s most expensive spice, is extracted from the dried stigma of Crocus flowers. Crocus flowers also contain several important pharmacologically active compounds, bitter principles (e.g., picrocrocin), volatile agents (e.g., safranal), and dyes (e.g., crocetin and its glycoside crocin). Active compounds have been used as anticonvulsant, antidepressant, anti-inflammatory, antitumor and neuroprotective agents. Crocin has also been reported to act as an anti-alzheimer agent by inhibiting pro-inflammatory activity, triggered by the microglia, and to have a beneficial impact on the cardiovascular systems, immune system, endocrine system, and the gastrointestinal tract [286]. Saffron (30 mg/day^−1^) has been used to treat mild to moderate depression in clinical outpatients with no side effects [287], and crocetin has been shown to have anti-proliferation effects on lung cancer cells in Swiss albino mice administered at 50 mg/kg^−1^ bodyweight [288]. It has also been used to suppress the proliferation of colorectal cancer cells in vitro (3 mg/mL^−1^) [289]. Crocin has been described as having an IC_50_ of 2mM in cervical cancer cell lines [290]. For an extensive review on the uses of saffron and other compounds, see Moshiri et al. [291], Milani et al. [187] and Pashirzad et al. [292] (Section 3.4; Figure 8).

#### 3.6.3. Carotenoid-Derived Ionones

β-ionone has also been described has having important pharmacological properties benefiting human health, including antibacterial [293], antifungal [293] and antileishmanial [294] activities. β-ionone actively inhibits *Escherichia coli* and *Candida albicans* proliferation [295] and the growth of the fungus *Aspergillus flavus* and sporulation of *A*. *flavus* and *A*. *parasiticus* [296]. β-ionone has also been shown to have cancer-preventing [297,298] and anti-inflammatory roles [299]. β-ionone has been shown to suppress the proliferation of breast cancer cells [298], prostate cancer cell growth in both in vitro and in vivo models [300] and induce apoptosis in murine B16 melanoma cells, human leukaemia and suppress the proliferation of human colon adenocarcinoma cell lines [301], human colon cancer [302] and human gastric adenocarcinoma [303]. Liu et al. [304] reported that β-ionone was responsible for a dose-dependent inhibition of mammary gland carcinogenesis in rats—further indication that ionones have important therapeutic uses (for review, see Ansari et al. [297] and Aloum et al. [305].)

Other ionones and their derivatives have also been shown to have therapeutic value. 3-Hydroxy-β-ionone, for example, was shown to slow the proliferation colony formation and cell migration of squamous cell carcinoma [306]. α-ionone derivatives have also been shown to exhibit anti-inflammatory, anti-microbial and anticancer effects. However, it appears that the use of ionones in therapy might be complicated by their interaction. For example, α-ionone prevented or suppressed the effects of β-ionone [307,308], and Neuhaus et al. [307] found that α-ionone inhibited the β-ionone-induced anti-proliferative effect in prostate cancer cells.

### 3.7. Apocarotenoids Have Roles in Plant Development and Defense

In addition to their roles as aroma, flavour and colourants, apocarotenoids have been shown to have a variety of functions in planta, including having roles in plant–microbe interactions, plant—insect interactions and in plant development.

#### 3.7.1. Apocarotenoids Promote Arbuscular Mycorrhizal Symbiosis and Have Antimicrobial Activities

The 9,10(9′10′) symmetric cleavage of diverse carotenoids by CCD1 results in the formation of a variety of C_13_ cyclohexone apocarotenoids, depending on the substrate, and rosafluene-dialdehyde (C_14_ dialdehyde) (Figure 4), corresponding to the central portion of the original carotenoid precursor [44]. Another route for the formation of C_14_ dialdehyde follows the cleavage of a C_40_ carotenoid by CCD7 or CCD4, resulting in a C_27_ apocarotenoid which is subsequently cleaved by CCD1 in the cytosol to form an addition C_13_ cyclohexone and rosafluene-dialdehyde (Figure 3) [190,228,250]. This C_14_ dialdehyde is thought to be the precursor of mycorradicin (10,10′-diapocarotene-10,10′-dioic acid), a yellow pigment that accumulates in the roots of plants infected with arbuscular mycorrhizal fungi [249]. Mycorradicin accumulates in the plastids in the roots and is stored as globules, which leads to changes in root morphology [309]. The accumulation of Mycorradicin seems to be associated with arbuscular mycorrhizal (AM) symbiosis [250,310]. The root symbiotic association of AM fungi (AMF) benefits the host plant by improving tolerance to biotic and abiotic stresses, mineral nutrition and impact plant developmental processes that effect root architecture flowering time, fruit and seed formation/quality [311,312,313].

Several C_13_ cyclohexone derivatives have also been identified in the same root tissue [249,310,314,315]. Application of blumenin (Blumenols), a C_13_ 3′-hydroxy cyclohexone carotenoid-derived product (likely derived from 3′-hydroxy-β-ionone; Figure 3) that accumulates in roots [249,314,316], strongly inhibits early fungal colonization and arbuscule formation, implying that cyclohexenone derivatives might act in the plant to control fungal spread [317]. Blumenols are classified into three groups: blumenol A, B and C. However, it is blumenol C glycosides that accumulate during mycorrhizal colonization, including in the roots of several plant species, i.e., tomato, barley and potato [318]. Wang et al. [318] also reported that blumenols accumulate in the shoots and leaves of plants with symbioses with arbuscular mycorrhizal fungi. These authors suggested that this accumulation may be useful, and potentially a universal indicator, of symbioses between different plants and fungi and that measuring blumenol levels in leaves, which would be quicker and simpler than trying to identify fungal symbioses in root soil samples, could be used by crop breeders to select cultivars that have better interactions with beneficial fungi (see [318] for review).

α-Ionone, derived from the 9′10 cleavage of α-carotene, inhibits the growth of multiple pathogenic fungi, including *Fusarium solani, Botrytis cinerea*, and *Verticillium dahliae* [319], *Colletotrichum musae* [320] and *Peronospora tabacina* [321]. β-ionone, derived from the 9′10 cleavage of β-carotene by CCD1/CCD4, has been shown to inhibit the sporulation and growth of *Peronospora tabacina,* a plant pathogenic fungus infecting tobacco [321,322]. Thus, it is possible that expression of CCD1A and CCD1B in vegetative tissues and fruit may have a role in the formation of multiple antimicrobial compounds.

#### 3.7.2. Apocarotenoids Attract and Repel Insects

β-Ionone has been shown to repel both the flea beetle and the spider mite and provide a significant oviposition deterrence to whiteflies [323]. Moreover, β-ionone (and geraniol (isoform of geranial generated by CCD1)) has been shown to repel the crucifer flea beetles (*Phyllotreta cruciferae* (Goeze)) from *Brassica napus* (L.) leaves [324] and conversely attract Euglossa mandibularis (*Hymenoptera, Apidae*) males [325], suggesting that it could be used in ‘push’ and ‘pull’ strategies for controlling pests in different crops dependent on the predominant pest (for review on β-Ionone, see Paparella et al. [262]). Geranylacetone has also been shown to attract Longhorn beetles (*Asemum caseyi*) and is a constituent, along with fuscumol, in traps used to attract a related Longhorn beetle, *Asemum nitidum* [326]. β-cyclocitral emissions from strawberries have been shown to attract spotted wing drosophila (*Drosophila suzukii* (Matsmura)), a pest causing damage to ripening fruit [327]. Furthermore, additional studies showed that males had higher responses to β-cyclocitral than females, suggesting that males have a greater sensitivity to this compound [328]. α-ionone induces tomato plant resistance to western flower thrips (*Frankliniella occidentalis*, see [329]) and MHO increases in wheat seedlings following infestation by the aphid *Rhopalosiphum padi*, repelling the aphid [330]. MHO is also released after infestation of the aphid *Uroleucon jaceae*, attracting a parasitoid wasp (*Aphidius ervi*) [331]. Vogel et al. [216] suggested that the activity of the insect would disrupt chloroplast integrity, exposing the CCD1 enzymes located outside of the chloroplast to the lycopene substrate localized inside, causing the rapid increase in MHO upon infestation.

The potential for engineering volatile production in specific plant tissues could be a viable strategy to repel pest and/or attract pest predators that could result in a reduced requirement for pesticides. The over-expression of AtCCD1 in Arabidopsis, for example, was shown to induce β-ionone emission [323,332], reducing feeding damage by the crucifer flea beetle, suggesting that the over-expression of CCD1 in crop plants could provide a natural repellent for some pests.

#### 3.7.3. Developmental Roles of Apocarotenoids

CDCs also play roles in plant development and plant defence. The most well-known CDCs, ABA and strigolactoes, formed by NCEDs and CCD7/CCD8, respectively, from neoxanthin (Figure 2) and β-carotene (Figure 3) are the most well studied. Other CDCs have also been shown to affect plant development. β-Cyclocitral, formed by the 7,8(7′8′) cleavage of β-carotene by CCD1/CCD4 activity, is an endogenous root compound that has been found to promote cell divisions in root meristems and to stimulate lateral root branching in Arabidopsis [333].

In *ccd1/ccd4* double mutants, β-Cyclocitral was shown to rescue meristematic cell division [333]. Application of β-cyclocitral to tomato and rice seedlings showed that it is a conserved root growth regulator across plant species resulting in a denser crown root systems in rice [333]. The positive effects of β-cyclocitral were also observed in plants grown in conditions of elevated salt and, and it was able to rescue rice roots, improving plant root depth and plant vigour [333]. This is consistent with the reports that β-cyclocitral mediates resilience to photooxidative stress [334,335] and initiates acclimation to high-light conditions [335]. Studies carried out in Arabidopsis have shown that β-cyclocitral acts as a messenger, conveying a singlet oxygen (^1^O_2_) stress signal to the nucleus, regulating the expression of ^1^O_2_ responsive genes [335,336]. A similar activity has also been described for dihydroactinidiolide, a volatile formed by the oxidation of the carotenoid derived β-ionone by singlet oxygen [335]. The accumulation of β-Cyclocitral in root tissue is consistent with the expression of CCD1 [40] and CCD4 [143] in tomato and potato roots, respectively (For review, see D’Alessandro and Havaux, [337]).

Furthermore, the symmetrical cleavage of lutein and zeaxanthin at the 9,10(9′,10′) positions leads to the formation of 3-hydroxy-β-ionone and 3-hydroxy-α-ionone (Figure 4). The 3-hydroxy-β-ionone (also formed by the 9,10(9′,10′) cleavage of zeaxanthin; (Figure 4). accumulates in etiolated bean seedlings on exposure to light. This compound may have a function in the light-induced inhibition of hypocotyl elongation [338,339]. Kato-Noguchi and Seki [340] showed that 3-hydroxy-β-ionone, produced by the moss *Rhynchostegium pallidifolium* (Mitt.), which typically forms large colonies on rocks and soils, inhibited the growth of *Lepidium sativum* L. (cress). Applied exogenously, 3-hydroxy-β-ionone was shown to inhibit the growth of hypocotyls (conc. 1 µM) and roots (conc. 1 µM) of cress [340]. These data suggest that 3-hydroxy-β-ionone plays a role in maintaining pure *R*. *pallidifolium* colonies by acting as a defence mechanism to suppress the growth competitors.

## 4. Future Prospects and Conclusions

Current estimates indicate that a >50% increase in the yield of most of the important food crops (wheat, rice and barley) will be needed to maintain food supplies by 2050. Furthermore, in order to tackle environmental changes, it will be necessary to breed and/or develop crop varieties with a higher nutritional quality to tackle what has become known as ‘hidden hunger’. In recent years, improving nutritional crops quality has become a target for supplementing the micronutrients in poor diets of remote communities where dietary variation is often limited (For a review on the dietary intake of carotenoids in different countries, see Meléndez-Martínez et al. [6] and references therein). Increasing both food resources and nutritional quality will require a multi-targeted approach touching on multiple aspects of plant development, including carbon assimilation and electron transport in leaves and non-foliar tissue (for review see [48,341,342,343]), light adaptation and water use efficiency [344,345,346,347,348] and biofortification [48,349]. Manipulating carotenoid biosynthesis (see Section 2.2.1) and carotenoid stockage (see Section 2.2.2), as discussed in this review, also adds the potential of improving the health benefits as well as the flavours and aromas of fruits and vegetables, potentially encouraging and promoting a more diverse and healthy diet.

Plant secondary metabolites have a high degree of nutritional and pharmaceutical potential which is still largely unexplored. These compounds have been used as medicines, biopesticides, bioherbicides and have been described as important to animal and human health. Many of them, both carotenoid (see Section 2.3) and their breakdown products (see Section 3.6), are reported to have anti-cancer and anti-inflammatory properties, to name but a few of their benefits. This knowledge allows us to target the breeding or engineering of crops with elevated levels of these compounds in conjunction with higher yielding varieties.

Knowing which enzymes generate many of these apocarotenoid also offers a significant opportunity to improve the health benefits and flavours of consumed products. For example, during processing, tomato pastes and sources are heated, which can result in the loss of many apocarotenoids due to their volatility. The over-expression and purification of CCD1/CCD4 recombinant enzymes (for example in food grade yeasts or bacteria) and their introduction into tomato pastes and other important food plant material (from pepper paste, avocado source, or fruit purees and juices) following processing aims to improve the flavour/aroma, quality and over-all fresh taste of these products. This could also result in a reduction in artificial flavour molecules added to foods, providing companies with a product with a more natural image. This would prove beneficial in the long term, allowing them to market a natural tasting and healthier product.

Agricultural research has adopted key technologies such as genetic engineering and genome editing to improve identifiable traits in crops [30,350,351], and new tools have been developed, including vectors multiple gene insertion [352,353,354,355,356] and tissue-specific promoters [99,357,358,359,360,361]. However, many countries still have an aversion to the use of these technologies, which are often complicated by non-science-based ideas [362,363,364]; both public perception and governmental bias around these technologies will need to be addressed, and a more streamlined, long-term approach to these technologies will need to be adopted.

## Figures and Tables

**Table 2:** Summary of the impacts of manipulating carotenoid accumulation by manipulating carotenoid storage sinks (Orange protein (Or); Fibrillin (Fib)) *Capsicum annum (Ca); Brassica oleracea (Bo)*. See Osorio et al. [136] for review.

Plant	Transgene	Metabolite Analysis	Ref
*Tomato fruit*	AtOr	Increases in Lycopene (1.6-fold), α-carotene 2.6-fold) and β-carotene (2.7-fold)	[129]
	CaFib	Increases in Lycopene (2.2-fold) and β-carotene (1.6-fold)	[22]
*Cassava tubers*	BoOr	~2-fold increases in carotenoids (as all-trans-β-carotene) (3–4 µg/g DW) compared to CN 0.5–1 µg/g DW)	[114]
*Sweetpotato*	IbOr	Total carotenoid levels (up to 7-fold) in their storage roots compared to wild type (WT). The levels of zeaxanthin were ∼12 times elevated, whereas β-carotene increased ∼1.75 times	[137]
*Potato tubers*	BoOr	Total carotenoid 6-old higher than CN. Increase from 4 µg/g DW to 22 µg/g DW	[128]
		Total carotenoids increased from 5.51 µg/g DW to 31 10 µg/g DW in the best lines, representing a 5.6-fold increase.	[131]
*Rice seed*	AtOr	Control rice seed contain no carotenoids. In conjunction with the over-expression of PSY and CrtI, Or expressing lines accumulated upto 25.8 µg/g DW total carotenoids (10.5 µg/g DW β-carotene)	[127]
*Maize seed*	AtOr	32-fold higher than wild-type controls ~25 µg/g DW	[133]

*Capsicum annum (Ca); Arabidopsis thaliana (At); Brassica oleracea (Bo);* CN = control.

## Data Availability

Not applicable.
